# An Exploratory Multi-Case Study of the Health and Wellbeing Needs, Relationships and Experiences of Health and Social Care Service Users and the People who Support them at Home

**DOI:** 10.5334/ijic.7003

**Published:** 2023-02-23

**Authors:** Louise Henderson, Heather Bain, Elaine Allan, Catriona Kennedy

**Affiliations:** 1 Bon Accord Care, Inspire Building, Beach Boulevard, Aberdeen, AB24 5HP, GB; 2 Robert Gordon University, School of Nursing & Midwifery, Garthdee Road, Aberdeen, United Kingdom, AB10 7QE, GB; 3 University of Highlands and Islands, Institute of Health Research and Innovation, Centre for Health Science, Old Perth Road, Inverness, United Kingdom, IV2 3JH, GB; 4 The Queens Nursing Institute Scotland, 31 Castle Terrance, Edinburgh, Scotland, United Kingdom, AH1 2EL, GB; 5 The University of Limerick, Department of Nursing and Midwifery, Limerick, Ireland, V94 T9PX, GB

**Keywords:** integrated health and social care, co-production, health and wellbeing needs, relationships or relationship-based care, community or communities, people-centred care

## Abstract

**Introduction:**

International policies and legislation set a precedence of person-centred sustainable integrated Health and Social Care (HSC) that meets the health and wellbeing needs of service users through improved experiences. However, current research focuses on service models, with fewer studies investigating experiences and needs.

**Methods:**

This qualitative multi-case [n = 7] study was co-designed with key stakeholders and aimed to explore experiences and needs of people who access and provide HSC at home. Data were collected in a regional area of Scotland (UK) via single [n = 10] or dyad [n = 4] semi-structured interviews with service users [n = 6], informal carers [n = 5] and HSC staff [n = 7] and synthesised using Interpretive Thematic Analysis.

**Findings:**

Interpersonal connections and supportive relationships were instrumental in helping all participant groups feel able to cope with their changing HSC needs and roles. They promoted reassurance, information sharing and reduced anxiety; when they were lacking, it negatively impacted upon experiences of HSC.

**Discussion:**

Promoting inter-personal connections that encourage supportive relationships between people who access and provide HSC and their communities, could promote person-centred Relationship-based care and improve HSC experiences.

**Conclusions:**

This study identifies indicators for improved HSC, advocating co-produced community-driven services to meet the self-defined needs of those who access and provide care.

## Introduction

Many populations across the globe are ageing, with growing numbers of people living with multiple long-term conditions, leading to increased complexity of care provision and rising demand for services [1]. Integrated Health and Social Care (IHSC) services offer a potential solution to support individual citizens across these populations [2]. Defining integration can be challenging, it can be seen as a design feature of service provision, organisational structures, or as a medium for delivering person-centred care (PCC) in an efficient way [3]. To add clarity to its context in this paper, IHSC is considered to be care that is delivered jointly between service users (people who use health and/or social care services), informal carers (people who offer non-contractual support to a service user), and health care and social care (HSC) organisations (including third sector and community initiative groups). Integration aims to promote greater simplicity in public services and facilitate timely, stream-lined access to appropriate HSC [4]. The actuality for some people who access HSC reflects services that do not always work together to provide care in an integrated way [5,6,7]. Moreover, despite widespread acknowledgement in the literature that people who use HSC should be involved in making decisions about their own care, they do not always feel as though they are [5]. Regional and local access to HSC can be variable, unequal and ill-suited to their needs, being disproportionate to the need and demand for services and reducing access to support [8,9]. There is a plethora of literature containing evidence-based accounts of assessing need, planning, implementing and evaluating IHSC models of care. Whilst these can guide HSC services and sectors in providing care, the experiences of service users can help to decipher health and wellbeing outcomes that are important to *them* [10,11]. However, there appears to be a paucity of evidence on the experiences of those who access and provide such services.

## Research design

### Involving stakeholders in this study

An integrative literature review was conducted as part of the lead authors PhD study. This review identified gaps in current knowledge about the experiences of people who accessed HSC. Findings were discussed with key stakeholders, including people who accessed and provided HSC services and members of the public. Those who accessed and provided HSC offered their verbal and written feedback in a series of three face-to-face roadshow events (April 2018 – Oct. 2019; attendance circa 80 people per event). Members of the public, who had expressed an interest in receiving information about research activity in their local area, also offered their verbal and written feedback in a community network group meeting (Dec. 2019; attendance circa 50 people). Their feedback and findings of the literature review informed the development of a short series of research questions (Table 1), aim and objectives (Table 2) for this PhD study. Their valued engagement, through early fieldwork and wider formal and informal engagement events, continued iteratively throughout this study, later converting to online engagement events under Covid-19 pandemic restrictions.

**Table 1:** Research questions.


RESEARCH QUESTIONS	

**Needs & Experiences**	What are the perceived health and wellbeing needs of HSC service users and the people who support them at home?

	What are the experiences of service users and the people who support them at home, when accessing or providing HSC?

**Relationships**	How do relationships, between service users and the people who support them at home, influence health and wellbeing and experiences of HSC?


**Table 2:** Key objectives.


RESEARCH AIM

To understand the health and wellbeing needs, relationships, and experiences of HSC service users and the people who support them at home (key stakeholders).

**RESEARCH OBJECTIVES**

1. To explore the health and wellbeing needs of key stakeholders in HSC.

2. To explore key stakeholders’ experiences of HSC.

3. To investigate how health and wellbeing needs influence experiences of HSC.

4. To explore relationships and connections between key stakeholders in HSC.

5. To investigate the significance of key stakeholder relationships on health and wellbeing.


### Study design and methods

Relationships between stakeholders in HSC, were explored using Yin’s [12] embedded model of multi-case study design and qualitative methods (Tables 1, 2). This design embraced each participant’s unique perspective whilst recognising a need for them to be ‘bound’ to others with whom they had a caregiver-receiver relationship.

#### Recruitment of contextual study sample

Scotland has an estimated population of 5,479,900, with 32 regional areas that have populations ranging from 626,410 to 22,190 [13]. Each regional area has one or more Health and Social Care Partnership (HSCP) areas within their geographical boundaries. These HSCPs facilitate operational delivery of an integration strategic plan to meet population health and wellbeing needs in their area [14].

An invitation to take part was distributed to potential participants in one regional area of Scotland with three HSCP areas, via professional social media accounts and a cascade email to HSCP staff. Service users [n = 6], informal carers [n = 5] and HSC staff members [n = 7] were recruited between September 2019 – February 2020. Participants [n = 18] were grouped in cases [n = 7]. A case was formed when a service user identified one or two people who supported them at home to take part with them.

**Figure 1:**
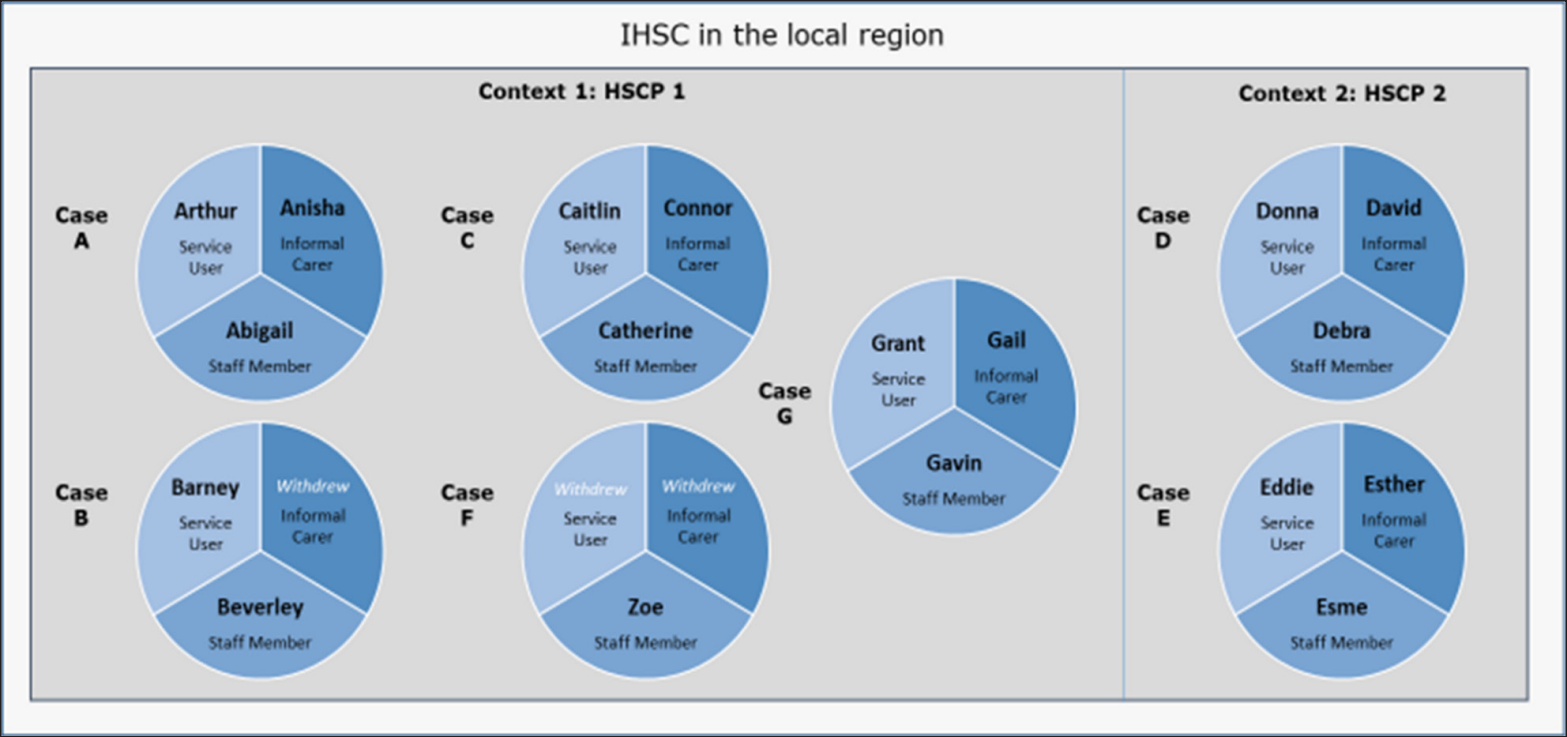
Contextual sample and cases.

Five cases had a service user, informal carer and staff member participant. One case contained one staff member participant, after the service user and informal carer withdrew from the study. One further case contained a staff member and a service user, when the informal carer participant withdrew. Cases were labelled A-F and participants were given pseudonyms to protect their identity (Figure 1).

#### Data collection

Data were collected via semi-structured interviews [n = 14] between Dec 2019 – March 2020. Service users chose an individual interview [n = 2] or a dyad interview [n = 4] with their informal carer. All Staff members [n = 7] and an informal carer [n = 1] were also interviewed individually. All interviews were conducted face-to-face, except for two individual telephone interviews [informal carer Esther; staff member Esme].

#### Data analysis

Simultaneous inter-case and cross-case analysis was conducted to explore the diversity of experiences and HSC needs across cases in the region [12,15]. Interpretive Thematic Analysis facilitated the development of a framework for developing insights, concepts and patterns of meaning across cases [12,15,16]. Researcher reflexive field notes, journaling, and ongoing review of the emerging findings promoted transparency and thick-description of methods, analysis and subsequent findings [15,17,18]. Analyses were reviewed by supervisors [n = 3] within the research team, and subject to ongoing external review via multiple research forums across both academic and HSC practice. Furthermore, members of the public in a community network group were invited to explore preliminary thematic findings during analysis, to iteratively develop interpretation of the data and subsequent findings [n = 12] (July 2021).

#### Promoting trustworthiness

Credibility, transferability, dependability and confirmability were promoted by incorporating multiple participant perspectives, pattern matching techniques, ongoing scientific review and involving stakeholders during data analysis, construction of themes and write-up [15,17,18]. Ethical approvals were gained in June 2019 at the hosting academic institution (SERP Reference Number: 19-12). Ethical approvals were granted in October 2019 by the UK-wide Integrated Research Application System (IRAS) (IRAS Project ID: 247771; REC reference: 19/NS/0148). They were granted with the IRAS Research Ethics Committee’s recommendation that people with learning disabilities and those with profound mental health issues were excluded (discussed further in the strengths and limitations section below).

## Key findings

### Context and overview of findings

To offer context to the experiences participants shared within their case, relevant background information has been included in Table 3.

**Table 3:** Relevant background information for participants and their cases.


CASE	PARTICIPANT GROUP	PARTICIPANT	PARTICIPANT/CASE BACKGROUND INFORMATION

**A**	Service user	Arthur	Arthur is retired and has early-onset Dementia. Although Arthur can move around independently, he struggles with sensory overload and impairment. He often forgets to attend to some of the functional tasks that help him to maintain his health and wellbeing (e.g., taking his medication, personal hygiene, maintaining an adequate fluid intake). He lives with his wife Anisha in the family home.

	Informal carer	Anisha *(Wife)*	Anisha is living with multiple long-term conditions and helps Arthur with prompting for medication, washing, dressing, meal and drink preparation and access to HSC services. They have two daughters who do not live near them and Anisha visits her daughters regularly whilst Arthur remains at home.

	Staff member	Abigail *(NHS Outreach Worker Dementia)*	Abigail supports Arthur and Anisha by offering information on his condition, helping them to access HSC services, and she reviews their needs regularly via ‘support visits’ at home, in outpatient clinics and on the telephone.

**B**	Service user	Barney	Barney is retired and is living with multiple long-term conditions. He lives in the family home with his wife. He experiences reduced mobility because of his long-term conditions and needs help with washing and dressing and administering prescribed creams. Members of his local community support him with regular social contact visits.

	Staff member	Beverley *(Carer)*	Beverley visits Barney daily to help him with washing, dressing and application of prescribed creams. She performs some housework tasks while she is there and collects prescriptions from the pharmacy for him.

**C**	Service user	Caitlin	Caitlin lives with her husband and her two teenage sons. She works part-time and has Multiple Sclerosis. Caitlin had an operation recently (unrelated to Multiple Sclerosis) and has found that she now needs increased help with housework tasks because of increased leg pain (unrelated to her surgery).

	Informal carer	Connor *(Husband)*	Connor is retired and lives with Caitlin in their family home, along with their teenage sons. He has been helping with housework tasks (Caitlin would have done these previously), and he helps her to access required HSC services (physio and General Practitioner.

	Staff member	Catherine *(Specialist Nurse)*	Catherine offers support to both Caitlin and Conner via outpatient clinics, telephone calls and home-visits.

**D**	Service user	Donna	Donna is retired and has Multiple Sclerosis. She lives with her husband David (semi-retired) in their family home. Donna is not able to stand unaided and needs the assistance of two people to help her transfer between bed and chair (Stand Aid or full body hoist). She has an automatic wheelchair which helps her to attend various hobby and interest groups independently. She uses public transport to attend these groups and goes to the shops independently in her wheelchair. Donna organises her own care through participatory budgeting. She employs carers to attend throughout the day to help with washing, dressing, toileting and transfers.

	Informal carer	David *(Husband)*	David helps his wife Donna in between carer visits (if needed) with meal preparation and empties her catheter bag. Occasionally he will help with washing and dressing if no carers are available. David has an adapted car that he and Donna use to go out together.

	Staff member	Debra *(Occupational Therapist)*	Debra has helped Donna and David, and Donna’s care staff, with monitoring health and safety, manual handling training and equipment. She maintains contact with Donna via home visits and telephone.

**E**	Service user	Eddie	Eddie is retired and has Multiple Sclerosis. He has recently moved into a sheltered housing complex where there is a resident warden. He mobilises independently with a three-wheeled trolley. Eddie socialises with others at the sheltered housing complex regularly. He walks to his local shop for social contact with the shopkeeper and to buy occasional-use small grocery items.

	Informal carer	Esther *(Daughter)*	Esther, lives in a nearby location to her father Eddie. She does not drive and takes the bus or gets a lift from friends to see Eddie every week. Esther works full time and helps Eddie with shopping, housework tasks and accessing required HSC services.

	Staff member	Esme *(Carer)*	Esme visits Eddie daily with other care staff who help him with washing and dressing, housework tasks (when required), meal and drinks preparation and catheter management.

**F**	Staff member	Zoe*(Befriender)*	Zoe is a Befriender who works for a voluntary organisation. She has been a Befriender for a ‘few years’ and shared her personal and voluntary experiences of HSC.

**G**	Service user	Grant	Grant is retired and lives alone in his own home. Members of his local community support him with social contact visits regularly, sometimes providing cooked meals. He has Chronic Obstructive Pulmonary Disease but can move about independently; however, he is limited in the length of time he can mobilise, because of shortness-of-breath linked to his condition. He drives to the local shop for a small number of groceries, but Gail helps him with larger amounts of shopping, and accessing required HSC services.

	Informal carer	Gail *(Daughter)*	Gail lives in a neighbouring area to her father Grant and visits him three times a week, helping with housework tasks. Gail submitted a request for a Befriender to visit Grant weekly, to ensure that someone had contact with Grant daily (Monday – Friday). A local cleaner has contact with him on the other day that Gail or the befriender does not visit.

	Staff member	Gavin *(Befriender)*	Gavin is a voluntary Befriender who has been visiting Grant once a week for two hours, for around two years. They talk about common hobbies, interests and family, and reminisce about historical events. Gavin does not help Grant with practical tasks, but he has offered to collect milk from the shops on his way to Grant’s house, on occasion.


**Figure 2:**
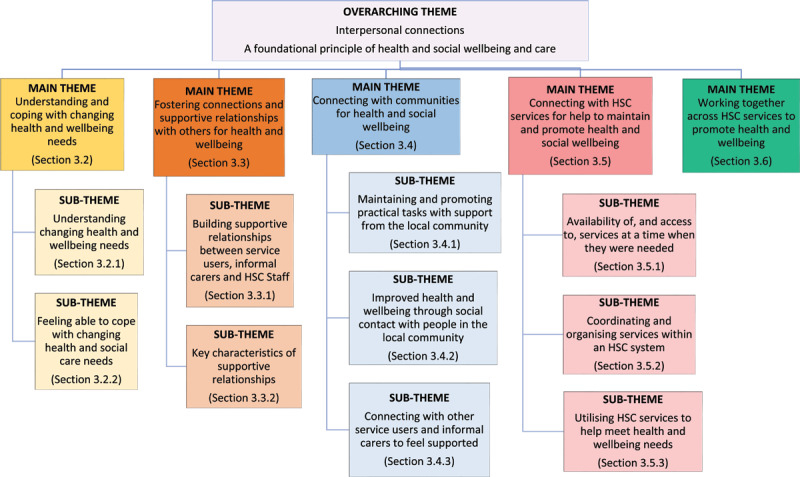
Overview of themes.

**Figure 3:**
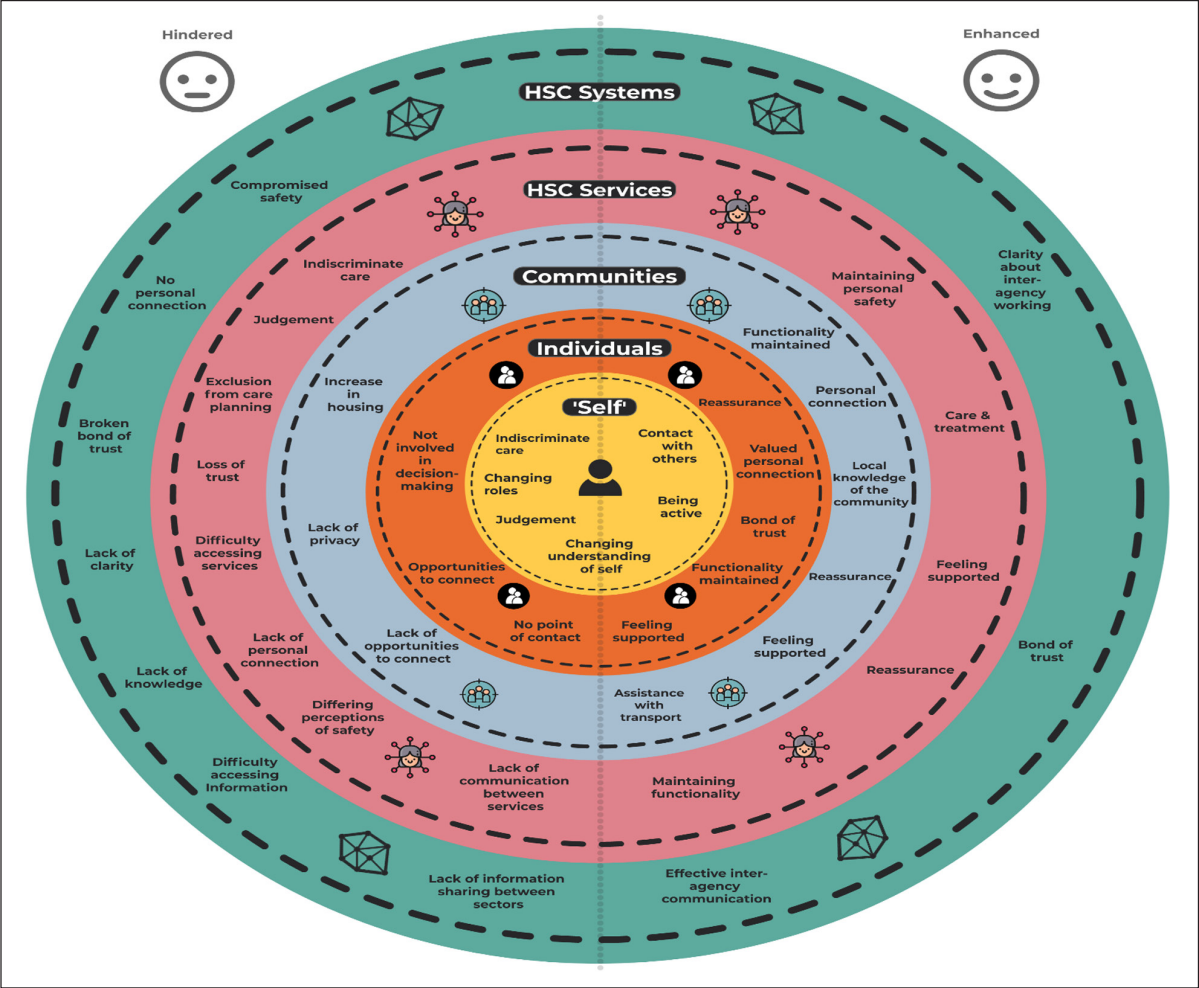
Factors that enhanced and hindered connections and experiences of HSC.

Following analysis, making interpersonal connections was identified as an overarching theme central to helping participants meet their health and wellbeing needs and/or those of others. Figure 2 presents five main themes representing the different contexts in which these connections were made, from understanding self, to linking with individuals, communities, services or wider systems. A summary of key factors that enhanced and hindered participants’ connections and experiences of HSC across these contexts is included in Figure 3.

### Understanding and coping with changing health and wellbeing needs

Participants across all groups discussed their experiences of understanding and coping with changing health and wellbeing needs or supporting others to do so.

#### Understanding changing health and wellbeing needs

Service user [n = 6] and informal carer [n = 7] participants’ understanding of their changing health and wellbeing needs, were shaped by their connections with others. They felt their experiences of accessing HSC could be challenging. Some HSC staff had focussed on service users’ medical conditions that were sometimes unrelated to their presenting complaint, suggesting some staff may not be adopting a person-centred approach to care:

*[Caitlin, quoting her Gastric Surgeon’s referral letter, gestures air-quotes] ‘Met with Caitlin, slim lady with Multiple Sclerosis, and I recommend that you give her a stoma’ [Catlin pauses, furrows brow and rolls eyes]*

*…that’s when I was an [gestures air-quotes again] ‘MS person’ and nothing else.”* ***Caitlin, service user, HSCP 1***.

All informal carer participants [n = 5] highlighted the impact their caring role had on their health and wellbeing, and their own need for person-centred support in their caring role. However, informal carer Anisha described an encounter with a Social Worker who exhibited a judgemental attitude, questioning her commitment as a wife and her role as an informal carer, when she raised the prospect of becoming unwell herself and the potential that she might not be able to care for Arthur:

*“The quote I got thrown back at me was, ‘a good wife would do that for her husband’, and I thought, yeah, if a good wife’s **here** type of thing but, I didn’t say it, I should’ve said it really or, maybe I should’ve just turned round and said, ‘well, I’m nae a good wife then!’, you know [crosses arms, frowns].” **Anisha, informal carer, HSCP 1***.

Informal carers [n = 5] also highlighted the negative impact that increased stress, as a result of their caring responsibilities, had upon their wellbeing. They reported low mood, depression, emotional strain and physical exhaustion. Staff member participants [n = 7] acknowledged their role in supporting service users and informal carers whilst their health and wellbeing needs were changing.

#### Feeling able to cope with changing health and wellbeing needs

Service user and informal carer participants emphasised a need for support from HSC staff with their changing health and wellbeing needs. They wanted reassurance and support from staff to feel empowered to make decisions about their care, and to access information to help them cope:

*“We get an appointment with them, just to go through things… they [HSC staff] don’t make up your mind for you but, they give the necessary information to allow you to come to a sensible decision… you canna make a decision on anything, if you don’t have the facts, you know.”* ***Arthur, service user, HSCP 1***.

For service users and informal carers, coping with changing health and wellbeing needs was also linked to being able to attend to practical tasks, such as managing finances, personal care, eating and drinking, managing continence, housework and shopping tasks. For some, getting ‘out and about’ to meet others [n = 3 informal carers] and contact with HSC staff [n = 4 service users], promoted engagement with communities and social contacts. However, all service user participants [n = 6] reported feeling lonely and isolated, and this was a source of concern for their informal carers [n = 3] and staff members [n = 2].

### Fostering connections and supportive relationships with others for health and wellbeing

Participants’ shared their experiences of fostering connections to build supportive relationships, which enhanced their health and wellbeing.

#### Building supportive relationships between service users, informal carers and HSC Staff

For all participant groups, connecting with others across HSC services, organisations and sectors to build a supportive relationship was facilitated through face-to-face interactions. Staff member [n = 4] and service user [n = 2] participants highlighted co-location of services as a means of promoting this. An interpersonal or ‘friendly’ connection was perceived by all participant groups as a necessary foundation for building supportive relationships. Commonalities between individuals was a key quality of these connections. Service user Donna, who had carers supporting her for a number of years, offers an example of this when she described her experiences of her need to connect with her HSC carer:

*“She’s [Donna’s Carer] chatting to me when I’m showering and I, I find out about her family and things, chat about her family and, you know, that sort of thing…*

*… I considered them as friends [her carers] and, I mean, I have a carer now, who’s been coming for over four years, in this company, and, I mean, she’s really efficient and, you know, I’m made to feel really comfortable and all that but, I mean, I said to her one day, do you think of me as a friend, or just another client [hesitates, looks down]… she [the carer] said, ‘well, just another client*’. ***Donna, service user, HSCP 2***.

During the interview, Donna’s non-verbal body language suggested she was disappointed with the disparity between the meaning her HSC carer had placed on the relationship and her own perceptions of it. For all participant groups, supportive relationships were fostered over a period of time, from a place of trust between two individuals, services or sectors. The opportunity to build up a trusting bond was afforded through continuity of contact between these groups, leading to a perception of more collaborative supportive relationships and HSC practices.

#### Key characteristics of supportive relationships

Key characteristics required for fostering a supportive relationship, as perceived by all participant groups, included personal attributes of empathy, trust, discernment and reliability:

*“I think Beverley works well because I can, I can count on her… I know that she’ll be there and that, that she won’t, you know, she won’t turn up sometimes and not others, that’s really quite important to me.”* ***Barney, service user, HSCP 1***.

Furthermore, when service user and informal carer participants were looking to foster a supportive relationship with HSC staff, they also wanted those staff to be knowledgeable about their condition and circumstances. All participant groups looked to share information, offer support and reassurance when communicating within supportive relationships. However, communication was perceived as challenging across HSC organisations and sectors. All participants [n = 14] attributed this to inefficient methods of communicating across organisational boundaries, with some staff members [n = 5] reporting system-wide data protection issues when trying to share information about those whom they were supporting.

### Connecting with communities for health and social wellbeing

Service user and informal carer participants’ experiences of connecting with communities helped them with practical tasks and to maintain social contacts, which were perceived to improve their health and wellbeing. Communities were defined by service user and informal carer participants as local geographical areas, meaning people who lived nearby. They also described communities, where people had a common interest or role such as a religious church group or a group of informal carers.

#### Maintaining and promoting practical tasks with support from the local community

Service users and informal carers reported that members of their communities provided valued reassurance and support. Some informal carers [n = 2] asked members of their communities to ‘check-in’ with their service user, and service users asked them to help with local grocery shopping and putting their rubbish bins out for collection [n = 2]. Connections that service users and informal carers had with people in their communities were often perceived as more cohesive than those they had with people from statutory HSC services. They attributed this to community members’ in-depth knowledge and understanding of their needs:

*“People that support me, are often people who are integrated into the local community so, people know them, erm, and they kind of know me so, that’s quite important to me, like… they know what I need and they, they know that I can’t walk too far so if, for example… I’d went and got some very heavy shopping; they would pick it up and put it in the car for me.”* ***Barney, service user, HSCP 1***.

However, for service user Grant, connecting with people in his community had become more challenging as local populations increased and neighbourly knowledge diluted:

*“The village is expanded so much, everybody before knew who I was and knew who the kids were but, no… you don’t know all the people now, you see, and there isn’t that contact, village contact, if you like… I don’t think it’s that open, er, neighbourly kind of care that used to be. The people probably are more dependent on, er, trained professional people.”* ***Grant, service user, HSCP 1.***

People in his community no longer had knowledge of his circumstances, leading to reduced informal support and to Grant feeling disconnected from his community.

### Improved health and wellbeing through social contact with people in the local community

Social contact with others had a positive influence on mental health and wellbeing. Previous knowledge of a service users’ circumstances helped community members to connect with them socially. All informal carers [n = 5] felt their service users’ social contact with others should be encouraged to promote mental wellbeing. Although service users wanted to maintain and make new social contacts [n = 4], when informal carer Esther encouraged service user Eddie to have social contact with others, he reminded her that he also needed time to himself:

*“He’ll [Eddie] sort of remind us, ‘I’m in my 70s! I actually quite like just sitting on my own sometimes and, like just having, having a wee [small] rest and taking it easy’.”* ***Esther, informal carer (talking about her father, service user Eddie), HSCP 2.***

This highlighted disparity between the expectations of some informal carers and service users, with relation to service users’ desire and need for social contact with others.

#### Connecting with other service users and informal carers to feel supported

Communities played a vital role in supporting service users, promoting connections and supportive relationships with people who knew and understood their circumstances. Maintaining and making connections with other service user and informal carers offered an opportunity for participants to share their experiences and access information about their condition or caring role, whilst offering peer support and social contact. However, not all service users and informal carers wanted to connect with communities of people who had similar circumstances or conditions [n = 2]. They reported anxieties around their future, and a risk of mis-matched expectations between treatment and progression of their condition:

*“My dad [Eddie] was sort of freshly diagnosed, he was sort of advised [by another person who also had Multiple Sclerosis] not to go along [to the support group], that he might find it a bit upsetting because there would be people there further along in the disease, in wheelchairs and really unwell. So, I think he sort of put off going”*. ***Esther, informal carer, HSCP 2.***

Service user participants who did attend these groups [n = 3], felt supported because they were able to exchange accounts of treatment options, discuss symptoms and disease progression, and connect with people who knew and understood their circumstances. However, it also presented challenges in other areas as highlighted through an anecdotal account from staff member Catherine:

*“I suppose it’s a great charity that patients, erm, get a lot out of [the support group], I’m sure. Sometimes their [the third sector organisation] opinions can be quite forceful, and we have to look at treatment options from an evidence-based practice [point of view] as opposed to perhaps what’s purported by the – [third sector organisation].”* ***Catherine, staff member, HSCP 1.***

### Connecting with HSC services for help to maintain and promote health and social wellbeing

Participants’ experiences of connecting with HSC services helped them maintain and promote their health and wellbeing, with availability, access, coordination and utility of HSC relevant to their needs.

#### Availability of and access to services, at a time when they were needed

Service user [n = 4], informal carer [n = 4] and staff member [n = 6] participants described their experiences of accessing HSC services, reporting fragmentation and reduced availability. It was important to all participants that service users and informal carers had timely access to services, such as physiotherapy and General Practitioners (GP). Service users [n = 6], informal carers [n = 5] and staff members [n = 7] reported reduced access to respite services, a need for greater flexibility in the way services were delivered, and reduced access to HSC services because of perceived obstructive ‘gatekeepers’:

“But there’s a woman in [location] who, you have to convince that you’re in need of the services.” ***Barney, service user, HSCP 1.***

Some service users [n = 3] and informal carers [n = 3] attributed reduced access and availability of services to financial constraints, and inflexible ways of working across HSC systems. Service user Barney perceived HSC as a ‘post-code lottery’, where services were available in some areas but not others and where the nature of individual HSC staff members influenced care. However, when participants could gain access to services at a time when they thought they needed them, they felt supported and that their health and wellbeing needs were being met.

“She [GP] gives us [Barney and his wife] such good support. It seems to me to be a bit of a lottery [access to a supportive GP], it depends very much on the nature of the, of the particular GP.” ***Barney, service user, HSCP 1.***

#### Coordinating and organising services within an HSC system

All participant groups were looking for further clarity on the way HSC was set up and organised across their services. Service users [n = 4] and informal carers [n = 5] perceived that having a named point-of-contact helped them achieve this:

*“Having a Care Manager [as a named point-of-contact] that, you know, coordinated things, that would refer you if you needed physio or OT or anything like that, that worked very well.”* ***Donna, service user, HSCP 2.***

However, not all service users had a point-of-contact. For some, this led to a perception of reduced levels of access to care and coordination. Service users and informal carers were not always involved in planning their own care, leading to them feeling disempowered. When they were involved in planning their care, they felt it was more efficient and timelier. They sought the support of HSC staff to plan for the future in order to ensure their changing health and wellbeing needs would be met. However, service user Arthur and his informal carer Anisha felt their Social Worker had demonstrated a short-term view and lack of pre-emptive planning of their care. Anisha explained that this made her feel as though she had fraudulently requested potentially unreasonable support; her non-verbal communication during the interview portrayed a sense of anger and distaste:

[crosses arms, purses lips, raises eyebrows and clicks tongue on the roof of her mouth] “It made me feel, almost fraudulent, as though I was asking for something that I shouldn’t have been asking for at that stage, or at this stage.” ***Anisha, informal carer, HSCP 1.***

#### Utilising HSC services to meet health and wellbeing needs

Participants’ use of HSC services to meet their health and wellbeing needs were varied. Some service users and informal carers were able to meet their needs through regular contact with a GP, specialist or wheelchair service. For some service users and informal carers, use of HSC was more challenging. They encountered lengthy waiting times and thought their care was not always appropriate, resulting in them using similar private sector services at their own expense.

“The waiting list is 15 weeks [for physiotherapy input], which isn’t handy if you can’t walk down the stairs! I couldn’t get away from it being sore… I think 15 weeks of that I would just be round the bend.” ***Caitlin, service user, HSCP 1***.

In addition, time constraints of support visits exerted negative pressure on service users and staff members relationships. For some staff members [n = 3], the relationships they formed with more experienced colleagues were instrumental in helping them to feel supported and boosting their confidence in their role.

### Working together across HSC services to promote health and wellbeing

Participants’ experiences of working together across HSC services and systems to promote and maintain health and wellbeing needs highlighted their understanding of ‘integration’. They acknowledged the positive effect that integration *could* have on HSC services, with pooled information and resources to promote better outcomes for service users and informal carers. However, ‘integration’ appeared to be an abstract concept to many participants (across all groups). Abigail reported a lack of clarity about structural changes, and a lack of communication and preparation for progressing them:

“Integrated HSC started up here maybe a couple of years ago, we were never really given a lot of information about it, naebody [nobody] ever came to speak to us about it and, to be quite honest, we’re nae [not] really sure how it’s supposed to work ‘cause naebody’s ever discussed it with us… I think a lot of our Locality Managers now are HSCP, as opposed to being health board.” ***Abigail, staff member, HSCP 1.***

Services were reported as disjointed, adopting unsafe communication and information sharing practices when bureaucratic processes did not meet the needs of HSC staff, the services or their HSC system. Some staff member participants [n = 6] suggested they needed further information about other HSC services working with people they were supporting. For staff member Debra, personal safety was compromised. She felt she had been placed at risk because of a communication breakdown that left her feeling vulnerable and uncomfortable:

“I had a patient who was very sexually inappropriate towards me, erm, I was just on my own in his house, so I called the Community Nursing staff to let them know [that the person had been inappropriate] but, they’d known about this for a long time and they had already made him double-visits [where two members of staff attend at the same time]. So, I’d been going in for months without knowing this, that was communication breakdown, it wasn’t nice what happened, and that could’ve been prevented had communication been that little bit better or, had we all been on the same system [electronic information system] and that would’ve flagged up for me. That would’ve saved a lot of uncomfortable feeling for myself [looks towards the floor, laughs uncomfortably, hesitates] … so.” ***Debra, staff member, HSCP 2.***

She felt that, had communication in the HSC system been better (through a joined-up electronic information system) this situation could have been avoided, mitigating risk for staff. Promoting trust between people within HSC systems helped to strengthen their supportive relationships and communication, and collaboration was achieved when people were experienced, knowledgeable and flexible in their approach to working with others.

## Discussion and theoretical contributions

**Figure 4:**
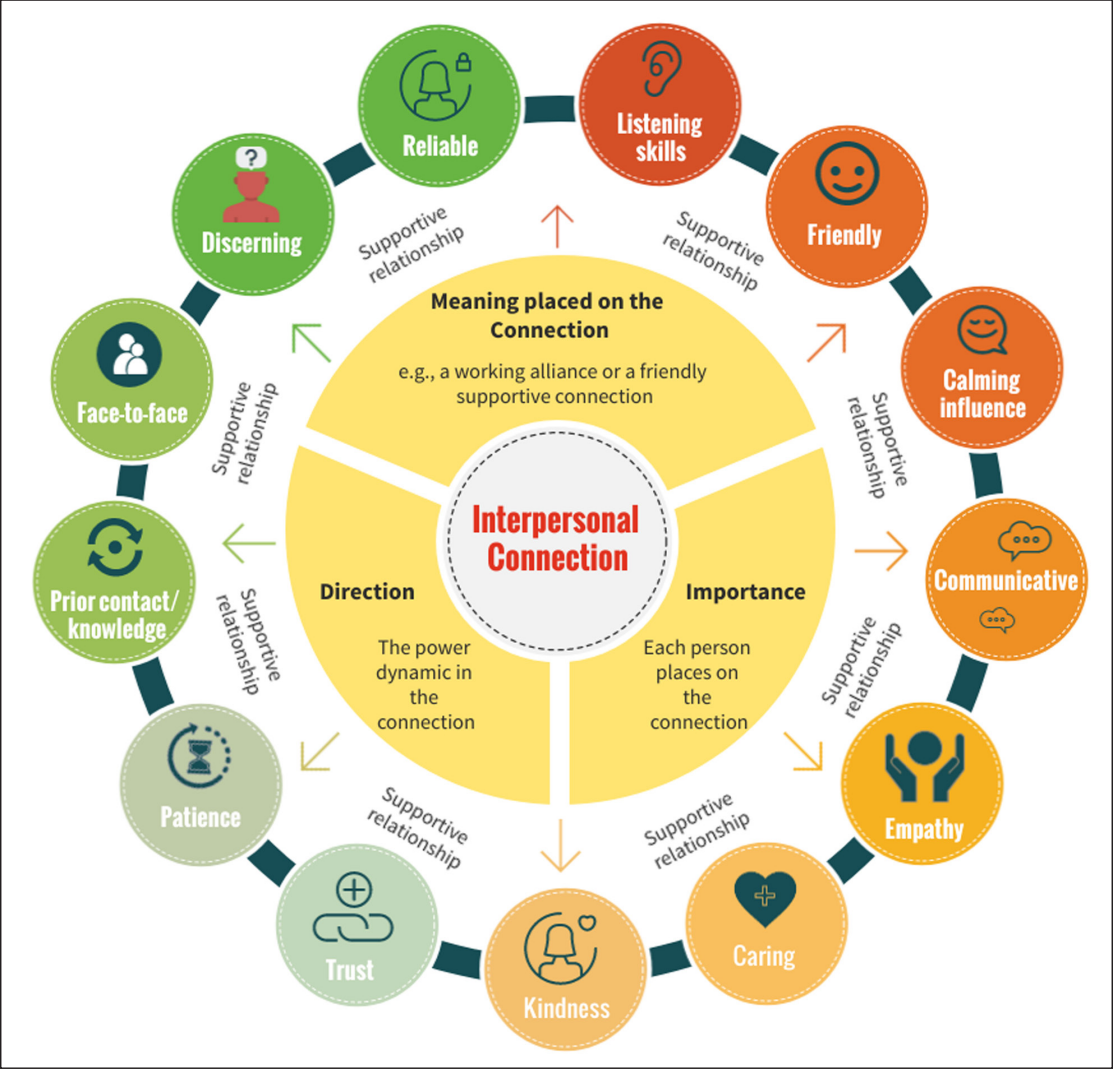
Typology of interpersonal connection and supportive relationships in HSC.

Findings of this study support the need for significant investment in facilitating and protecting the allocation of HSC staff time to help them develop supportive relationships with service users, informal carers and other staff across HSC systems. This is based on the understanding those in the relationship maintain contact or interaction over a period of time through continuity [19,20,21]. Participants highlighted several key characteristics that they perceived as important in interpersonal connections and supportive relationships, which are represented in a typology below (Figure 4).

In addition to the key characteristics of interpersonal connections and supportive relationships, participants across all groups outlined what they perceived as their health and wellbeing needs (Table 4).

**Table 4:**
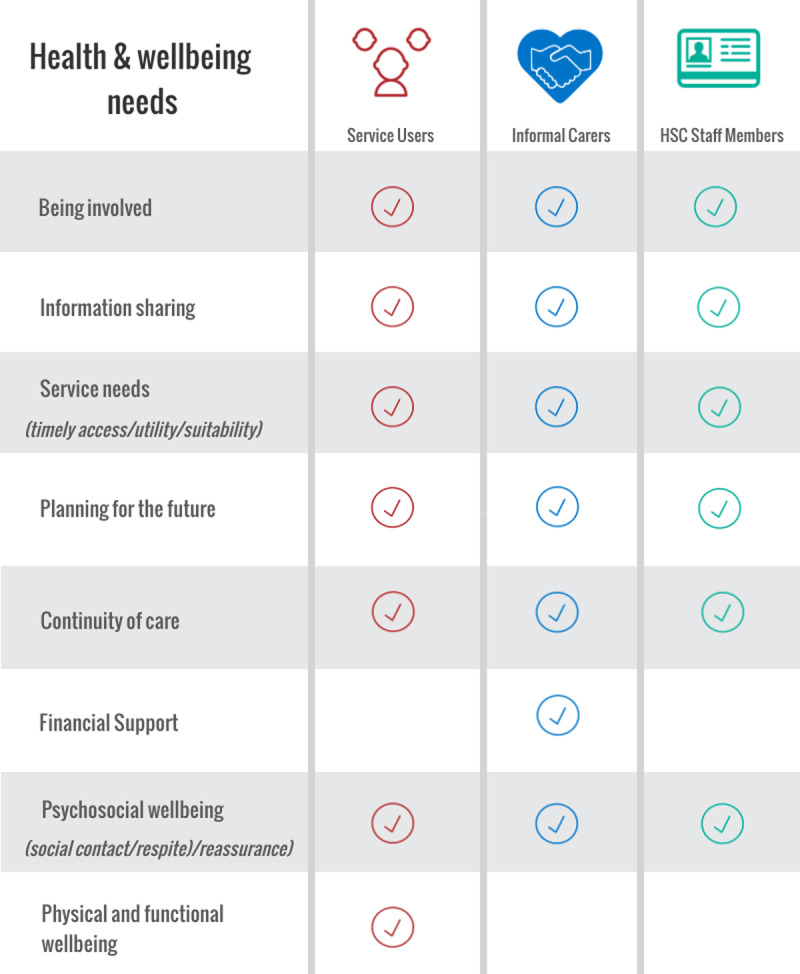
Health and wellbeing needs in HSC.

Findings of this study suggest models of HSC should promote the principles of interpersonal connection outlined above, and encourage supportive relationships between service users, informal carers and HSC staff members as a foundational principle of HSC. The concept of connection, through continuity of contact with someone who offers support, has long been acknowledged in relevant models and frameworks as a fundamental principle of PCC for people who access HSC services. Continuity has been advocated across a variety of contexts for many years, to help reduce admissions to hospital, lower HSC costs, and promote Service User and staff satisfaction [22,23]. Findings of this study support a significant investment in facilitating and protecting the allocation of HSC staff time; it can help them to develop supportive relationships with service users, informal carers and other staff across HSC systems.

**Figure 5:**
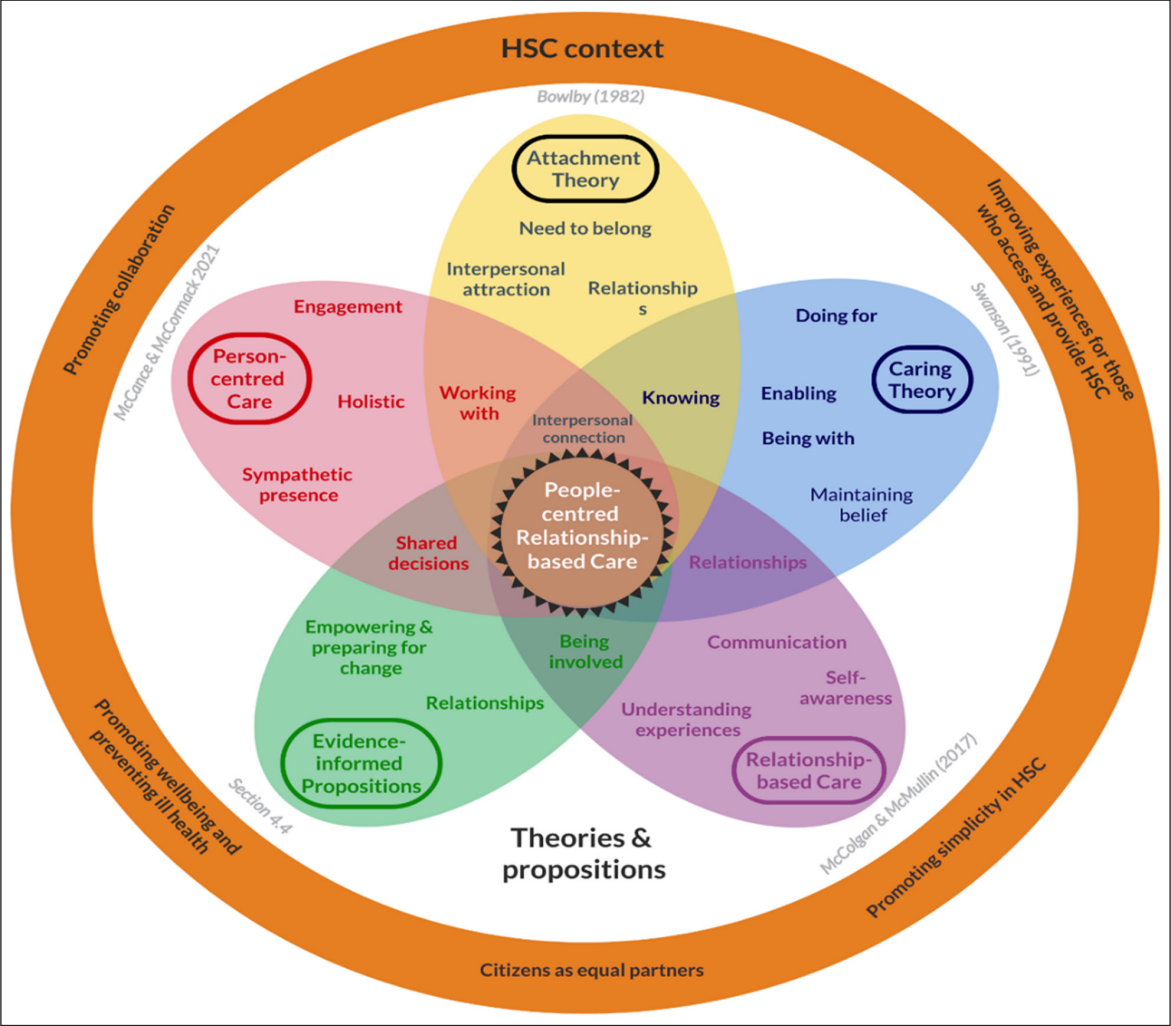
Theoretical and contextual influences: People-centred Relationship-based Care.

Many existing frameworks, theories and concepts identify key principles for integrating, improving and delivering HSC and PCC [5,24]. Some key theories were considered when interpreting participants experiences in this study. It is suggested that the findings outlined add to these. When interpreting participants’ experiences of fostering connections and relationships in a care provider-receiver context, behaviours linked to applications of Bowlby’s Attachment theory across the lifespan, were instrumental [25,26,27]. To further acknowledge the influence of connection in an HSC environment, a ‘blended’ theoretical lens was adopted. Caring Theory [28], Person-centred Care [29], Relationship-Based Care theories [30] and evidence informed propositions about experiences of people who access HSC [5] were combined. Figure 5, blends these key theoretical constructs and contextual influences that were important to study participants.

These theories and propositions intersect as People-centred Relationship-based Care, reflecting the key concepts of ‘integrating HSC’, as identified by the study participants: people, services and systems being ‘connected’ through supportive relationships; encouraging knowledge and understanding between people who access and provide HSC; being involved in making decisions about their own care or role, and working together to meet a shared desire for truly individualised care.

## Key learning and application to HSC practice

**Figure 6:**
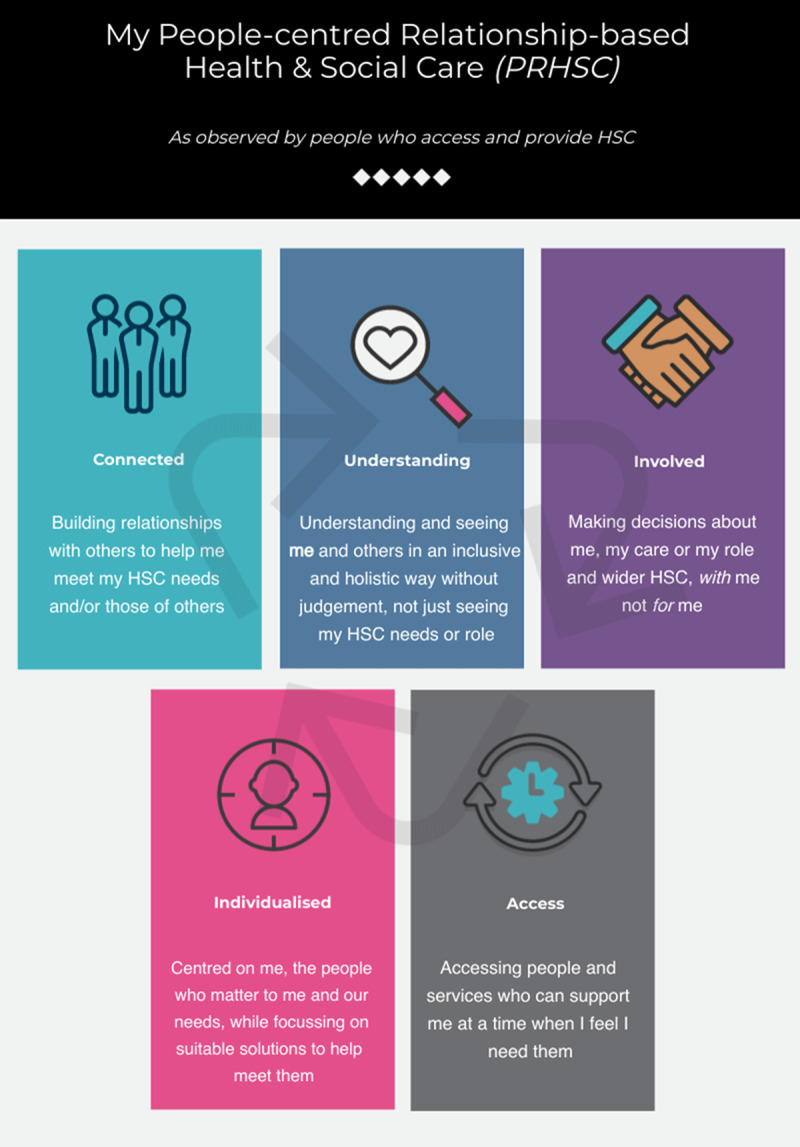
My People-centred Relationship-based Health and Social Care (PRHSC).

In an online engagement event, members of the public, HSC service users, and informal carers, offered their insights on how this study’s findings might be applied to HSC practice in their local areas [n = 12] (July 2021). They were clear that they wanted their services to provide streamlined and holistic HSC, regardless of organisational or sectorial boundaries. The concepts of People-centred Relationship-based Care have been framed from the viewpoint of participants and entitled ‘My People-centred Relationship-based Health and Social Care’ (PRHSC) (Figure 6).

The PRHSC model and its underpinning theories add an original perspective to key concepts of integrating HSC, as perceived explicitly by the people who are at the very centre of accessing and providing it (service users, informal carers and staff members). Furthermore, it could be argued that it could complement existing models of IHSC, for example, the International Foundation for Integrated Care’s nine key conceptual ‘Pillars of Integrated Care’ [31,32]. The PRHSC model’s underpinning blended theories (Figure 5) align closely with fundamental human rights, contributing to social justice by promoting equality and inclusion [33]. They highlight key insight into participants’ perceptions of integrated care in HSC practice. The application of the PRHSC model should be tested across different groups of people who access HSC in a variety of settings, to establish reliability and the viability of its use. Further exploration of the potential transferability of findings beyond HSC would be warranted across wider communities. For example, industries or public service sectors where elements of caring are incorporated, such as policing or education. It could be argued that these communities may also benefit from a deeper understanding of interpersonal connections and supportive relationships to inform the caring elements of their work.

## Strengths and limitations

The active part that members of the public and key stakeholders played in developing ideas, study design and refining interpretations, is key to the credibility of these findings. A further strength is its multi-case embedded design, which allowed multiple perspectives of participants’ reality to be represented in the data within each case. Reflexivity incorporating reflection, curiosity and consultation with key stakeholders and the research team, underpinned the entire research process thereby increasing the trustworthiness and transferability potential of these findings.

A potential limitation to the transferability of these findings is the contextual nature of HSC with the study population being from two HSCPs in one region in Scotland. In line with the ethics panel recommendations (Section 2.2.4), people who have learning disabilities or profound mental health issues were excluded from this study. This is recognised as a limitation and including these communities could have added depth of understanding and promoted relevance to wider practice areas [34,35,36].

## Conclusion

The overarching purpose of this study was to explore and better understand the health and wellbeing needs, experiences and relationships of people who accessed HSC and the individuals who supported them at home. Interpersonal connections that developed into supportive relationships were perceived by participants in all groups as instrumental in helping them feel able to cope with their changing HSC needs and roles. Supportive relationships promoted reassurance, information sharing and reduced anxiety; when they were lacking, it negatively impacted upon their experiences of HSC. This study highlights that connections, relationships and cross-sectoral working are important and entirely necessary for integrating HSC services. It is important that policy makers and HSC providers recognise the contribution communities can make to HSC; those communities come in many forms, and one model of integrating HSC does not fit all. No one person or service can provide the whole care-package, and all those who access and provide HSC need to have an equal voice. If the integration of HSC is to be improved, we as a society, must be clear on what is expected of HSC services and systems, and how we prioritise the limited resource across all contexts of HSC to meet health and wellbeing needs.
